# Relaxation of the Perineum and Vulva during Confinement

**Published:** 1886-11

**Authors:** Edward B. Angell


### *RELAXATION OF THE PERINEUM AND VULVA DURING CONFINEMENT.*

By Dr. AUVARD.

Translated from the *Journal de Médecine*, by Edward B. Angell, M. D.

In order to allow the passage of the fœtus, the soft parts which form the genital canal have to dilate and open, without which delivery would be impossible. This relaxation during labor is manifested successively in the course of the cervix, of the vagina and perineum, and finally of the vulva. The only question here considered relates to the perineum and vulva, the soft parts which require special attention on the part of the obstetrician, for they frequently are the cause of a difficult labor, while after confinement their lesions may have serious effect upon the health of the woman. Relaxation of the perineum and vulva may be accomplished in three different ways: by the bag of waters; by the presenting part of the fœtus; or, finally, by manipulation.

*Relaxation produced by the Bag of Waters*:—The bag of waters is, as we know, that part of the envelopes of the ovum which is exposed by the dilatation of the cervix. Under normal conditions the bag of waters is ruptured spontaneously when the dilatation is complete. It is through this opening that the fœtus passes from the interior of the ovum and the genital organs. In some cases this rupture is delayed, and then the bag of waters, in accordance with the advance of labor, protrudes more and more perceptibly. It encroaches upon the vagina, reaches the orifice of the vulva and forms a hernia. These cases of tardy rupture have been attentively studied by Dr. Byford of

Chicago. This celebrated accoucheur believes that in a similar way the bag of waters acts efficiently upon the perineum and the vulvo-vaginal opening and occasions their distension. The action of the bag of waters is the same upon the soft vulvo-perineal tissues as upon the cervix, whence the conclusion that this membrane should not be ruptured artificially, till dilatation is complete. In accordance with this theory, Dr. Byford recommends, in cases where rupture of the membranes occurs before this distension of the orifice is accomplished, the introduction within the vagina of a rubber bag, which may be inflated with air or water. In reply to Dr. Byford, Dumas discusses the following question : Is it possible in the majority of cases, to count upon the bag of waters for dilating the lower orifice of the vagina? And, at first, how is the bag of waters formed? Authorities are scarcely explicit upon this subject. Two hypotheses are held at present: first, either the membranes undergo an enormous distension at the level of the cervix, thus causing the prolongation which forms the bag of waters (theory of elongation) ; or, secondly, the membranes, up to a certain height, are separated from the lower segment of the body of the uterus, and come into contact with the obliterated cervix (theory of detachment or gliding). Against the theory of elongation the experiments of Duncan may be cited, which show that the membranes are very slightly elastic. The more rational theory is the one of detachment and gliding. The ovum is detached from the inferior segment of the uterus, and the uterine wall, owing to the obliteration and dilatation of the cervix, is drawn up along the wall of the ovum leaving it partially exposed. It is not, according to Dumas, the ovum which slides down along the uterine wall, but rather the uterine wall which glides up over the ovum. The ovum is passive, the wall of the uterus active. By this mechanism of detachment are readily explained the slight hæmorrhages which are noticed at the approach of labor, as well as placental detachment in cases of placenta prævia. With this detatchment, which may be very extensive, we can understand the possibility of a very prominent bag of waters without needing to assume great elasticity of the membranes. Why is it that, with the freedom given to it by this slipping upward, the bag of waters, is so flattened in normal cases, in normal presentations of the head? This is due to an interesting mechanism. The head forms a plug, when applied upon the inferior segment of the uterus, in such a way that the amniotic fluid above does not escape. Hence, at the moment of contraction, the fluid beneath the head is subjected to a relatively small pressure, in consequence of which there is little or no bulging of the membranes.

When the dilatation is complete, the cervix no longer sustains the head which descends and, while pinning down the membranes laterally, presses upon the bag of waters in such a way that rupture takes place at this moment. In cases of faulty presentation, the fœtal part not forming a cork, the amniotic fluid may accumulate in the bag of waters, which then becomes enormous. Hence it is plain that in normal presentations of the vertex, a voluminous bag of waters cannot exist, and to attempt to gain this object, as Dr. Byford advises, for the purpose of facilitating distension of the vulvo-vaginal orifice, is an impracticable matter. M. Dumas shows clearly that a voluminous bag of waters is, or ought to be, an exception, but he does not assert whether, in the cases where it does exist, it is capable or not of dilating the vulvo-vaginal opening. Now, in the different cases where I have seen this special form of the bag of waters, I have not observed it exercise any real influence upon the external organs of generation. Upon the cervix itself the bag of waters only acts efficiently when it is accompanied and reinforced by a fœtal part. It is very probable that alone it is powerless to open the uterine gate. Call to mind those enormous bags of water filling the whole vagina while the uterine orifice is hardly open. It is plain that if the suggestion of Byford is not practicable, it little matters, for, if it were possible, delivery would in no way be facilitated.

*Relaxation produced by the Fœtus:*—Relaxation of the perineum and vulva is produced in the natural way by the fœtal part, which, driven onward by the utero-abdominal contractions gradually opens a way of escape. Through this effort the dilatation of the soft parts is accomplished. The different fœtal regions are not equally adapted to this function, and for the same presentation, position is not a matter of indifference. For example, in a head presentation the distension of the soft parts is better accomplished with an occipito-pubic than with an occipito-sacral position. It is for the sake of remedying the difficulties of this stage of labor that special manipulations have been employed to which a few words will be devoted.

*Relaxation produced by Means of Manipulation:*—For a long time obstetricians have attempted various measures for distending and opening the soft tissues of the genital canal along which the fœtus escapes, hoping thus to facilitate labor. These attempts have been directed either to the os uteri, the vagina, or the perineum and vulva; they are known by some accoucheurs under the name of the "lesser labor" in contra-distinction to the real labor, the former being artificial, while the latter is natural. The most of these measures have to-day fallen into disrepute, for not only are they nearly always useless, but they may become mischievous through causing irritation and inflammation of the maternal tissues. It is necessary to remember that if this conclusion is well founded with respect to the cervix, it is not wholly so regarding the vulvo-vaginal orifice. Among the various expedients resorted to for this purpose, one of the best, certainly, is that suggested by Prof. Dumas and published in the *Montpellier Médical* of 1883, to which he gave the generic name of præ-fœtal dilatation of the vulva. The *modus operandi* is as follows: Three fingers of the right hand (the thumb, index and middle finger) are, at the moment of expulsion of the head, introduced into the vagina. Applied upon the fœtal head they are gently separated so as to make them glide between it and the vaginal orifice. They should be applied deeply enough to experience a moderate degree of pressure between the head and the vaginal opening. In this manner the fingers form in front of the head a sort of a cone or tripod, which passes through the vulva, its base being applied to the head, while its pointed end is outside. This cone, which thus surmounts the fœtal head in a way similar to a clown's cap, exerts a preliminary dilatation upon the vulvo-vaginal orifice. The influence of the bag of waters upon the cervix, as Dumas suggested, may be compared to the artifice resorted to by pharmacists in passing a rubber ring about the neck of a bottle; a small wooden cone is placed over the cork, point upward, along which the elastic ring glides, gradually enlarging as it is pushed downward.

M. Dumas denies any sort of relationship between his method and the "lesser labor" of certain obstetricians. In the "lesser labor" the fingers act directly upon the soft tissues, producing relaxation by means of massage or stretching. In præ-fœtal dilatation it is the fœtal head itself which acts upon the vulva through the intervention of the fingers. There is, perhaps, between the two methods a shade of difference, but only a shade.

The clinical results obtained by præ-fœtal dilatation have been stated by M. Passarini in his inaugural thesis. Out of sixteen cases where the method had been employed, twice only was there a tear of the perineum, of 1 ¼ inches in one case, and 1 ½ inches in the other. Præ-fœtal dilatation hastens markedly the stage of expulsion, which, in place of lasting two to three hours, is accomplished within the limits of twenty-five minutes to an hour and twenty minutes.

*Conclusions* :—What practical deductions may be drawn from what has preceded? Should a physician, attending a woman in confinement, rely upon the bag of waters and upon the fœtal portion presenting to effect the dilatation of the perineum and the vaginal orifice, or should he endeavor to give assistance by means of manipulation? We have only a choice between natural distension, spontaneously effected by the fœtus impelled by the utero-abdominal contractions, and the method of præ-fœtal dilatation. Now, we believe the accoucheurs of Paris are pretty unanimous upon this question. Save some exceptional cases where such measures may be safe, and among them that of M. Dumas is certainly one of the best, it is preferable to leave to natural forces the distension of the soft parts of the mother, the attendant combating, on the one hand, over energetic contractions of the uterus and abdominal walls, and giving assistance, on the other, by the application of forceps or by manual traction, if their action is too feeble. With the eminent professor of Montpellier we believe one can never reckon upon the bag of waters, its action being eminently serviceable for dilating the os uteri, but inefficient upon the perineum and vulvo-vaginal orifice.

The duty of the accoucheur is to sustain the perineum, not to dilate, depress or open it. Either the perineum is sufficiently elastic and the fœtus depresses it without tearing it, or its elasticity is deficient, and all methods are powerless to give it that suppleness essential to normal delivery. Every measure, which consists either in drawing the perineum forward, in pushing it backward or in an effort to give it tone by stretching the lateral portion of the vulva, seems to us equally illusory. The secret of the integrity of the perineum, during vaginal delivery, may be summed up in two principal indications; first, to retard the progress of the presenting portion of the fœtus; second, to direct this fœtal part in its adaptation to the form of the genital canal.

